# What influences 11-year-olds to drink? Findings from the Millennium Cohort Study

**DOI:** 10.1186/s12889-016-2847-x

**Published:** 2016-03-04

**Authors:** Yvonne Kelly, Alice Goisis, Amanda Sacker, Noriko Cable, Richard G. Watt, Annie Britton

**Affiliations:** Department of Epidemiology and Public Health, University College London, London, WC1E 6BT UK; Department of Social Policy, The London School of Economics and Political Science (LSE), Houghton Street, London, WC2A 2AE UK

**Keywords:** Alcohol, Early Adolescence, Millennium Cohort Study, Parent Drinking, Peer Drinking

## Abstract

**Background:**

Drinking in youth is linked to other risky behaviours, educational failure and premature death. Prior research has examined drinking in mid and late teenagers, but little is known about the factors that influence drinking at the beginning of adolescence. Objectives were: 1. to assess associations of parental and friends’ drinking with reported drinking among 11 year olds; 2. to investigate the roles of perceptions of harm, expectancies towards alcohol, parental supervision and family relationships on reported drinking among 11 year olds.

**Methods:**

Analysis of data from the UK Millennium Cohort Study on 10498 11-year-olds. The outcome measure was having drank an alcoholic drink, self-reported by cohort members.

**Results:**

13.6 % of 11 year olds reported having drank. Estimates reported are odds ratios and 95 % confidence intervals. Cohort members whose mothers drank were more likely to drink (light/moderate = 1.6, 1.3 to 2.0, heavy/binge = 1.8, 1.4 to 2.3). Cohort members whose fathers drank were also more likely to drink but these estimates lost statistical significance when covariates were adjusted for (light/moderate = 1.3, 0.9 to 1.9, heavy/binge = 1.3, 0.9 to 1.9). Having friends who drank was strongly associated with cohort member drinking (4.8, 3.9 to 5.9). Associated with reduced odds of cohort member drinking were: heightened perception of harm from 1–2 drinks daily (some = 0.9, 0.7 to 1.1, great = 0.6, 0.5 to 0.7); and negative expectancies towards alcohol (0.5, 0.4 to 0.7). Associated with increased odds of cohort member drinking were: positive expectancies towards alcohol (1.9, 1.4 to 2.5); not being supervised on weekends and weekdays (often = 1.2, 1.0 to 1.4); frequent battles of will (1.3, 1.1 to 1.5); and not being happy with family (1.2, 1.0 to 1.5).

**Conclusions:**

Examining drinking at this point in the lifecourse has potentially important public health implications as around one in seven 11 year olds have drank, although the vast majority are yet to explore alcohol. Findings support interventions working at multiple levels that incorporate family and peer factors to help shape choices around risky behaviours including drinking.

## Background

Regular heavy and binge drinking are recognised as major public health problems in terms of mortality, morbidity and wider social and economic consequences [1, 2], and regular and heavy drinking in youth are related to risky behaviours, educational failure and to the leading causes of death in adolescence [3–5]. Among the vast majority of people who consume alcohol, initiation of drinking takes place during adolescence [6]. The question remains open as to whether early initiation of drinking causes problematic alcohol use later in life with recent review articles reaching opposing conclusions [7, 8]. However, the importance of adolescent drinking is likely shaped by the timing and pattern of drinking as well as the broader social context. Research from Italy and Finland suggests the significance of context specific alcohol socialisation processes in relation to adolescent drinking [9]. Over the last decade there has been a decline in the prevalence of drinking among adolescents in the UK [10], however consumption levels among UK youth remain higher than the European average [11]. Among UK adolescent drinkers there is no evidence of a reduction in the quantity of alcohol consumed [10], and hospital admissions due to alcohol among the under 18 s remain a concern [12].

Adolescence is a time of dramatic change that influences young people’s sense of autonomy and their exploration of risky behaviours. Factors shown to influence young people’s drinking include parent and peer drinking behaviours, perceptions of risk, expectancies towards alcohol and supportive family relationships [13–17]. Most prior studies have focused on drinking behaviours in mid and late teenage years [13, 17–19] and as highlighted in recent reviews [4, 20] less is known about influences on drinking among pre-teens. Improving our understanding of factors that influence drinking initiation at the beginning of adolescence could help develop policies and effective alcohol harm reduction strategies.

Given the paucity of work on the initiation of drinking in very early adolescence, in this paper we address two research objectives 1. to assess associations of parental and friends’ drinking with reported drinking among 11 year olds; and 2. to investigate the roles of perceptions of harm, expectancies towards alcohol, parental supervision and family relationships on drinking among 11 year olds. To do this we analysed data from the large contemporary population based Millennium Cohort Study.

## Methods

The Millennium Cohort Study (MCS) is a UK nationally representative prospective cohort study of children born into 19244 families between September 2000 and January 2002 [21]. Participating families were selected from a random sample of electoral wards with a stratified sampling design to ensure adequate representation of all four UK countries, disadvantaged and ethnically diverse areas. The first sweep of data was collected when cohort members were around 9 months and the subsequent four sweeps of data were collected at ages 3, 5, 7, and 11 years. At the 11 year sweep, interviews were conducted during home visits with cohort members and their carers, and questions asked about alcohol consumption, socioeconomic circumstances and family relationships. Cohort members filled out a self-completion booklet in a private place within the home. Interview data were available for 69 % of families when cohort members were aged 11.

### Drinking at age 11

In a question developed for the MCS survey, cohort members were asked “Have you ever had an alcoholic drink? That is more than a few sips?” (yes/no).

### Parent and friends’ drinking

Parents were asked about the frequency and amount of alcohol they drank. “How often do you have a drink that contains alcohol?” (4 or more times a week, 2–3 times a week, 2–4 times per month, Monthly or less, Never). “How many standard alcoholic drinks do you have on a typical occasion?” Response options on frequency and quantity of alcohol consumed meant it was only possible to approximate drinking categories as set out in guidelines by the UK Department of Health. The same categories were used for mothers and fathers as follows: None; Light/moderate - those who drank but were not heavy/binge drinkers; Heavy/binge - 4 or more times a week and drinks a minimum of 3–4 drinks per drinking occasion, or a minimum of 5–6 drinks per occasion. Separate categories were created for cohort members where information on parents’ drinking behaviour was missing and when the father was absent from the household.

Friends’ drinking was assessed by asking cohort members “How many of your friends drink alcohol?” Response categories were recoded: None of them as No; Some/Most/All of them as Yes; don’t know was retained as a separate category.

### Covariates

#### Cohort member and family characteristics

Gender; puberty, assessed from responses by the mother to questions (for girls - hair on body, breast growth, menstruation, boys - hair on body, voice change, facial hair); birth order (first vs subsequent); current socioemotional difficulties (normal vs high score) [22]; antisocial behaviours (“Have you ever … been noisy or rude in a public place so that people complained or got you into trouble? … taken something from a shop without paying for it? … written things or sprayed paint on a building, fence or train or anywhere else where you shouldn’t have? … on purpose damaged anything in a public place that didn’t belong to you, for example by burning, smashing or breaking things like cars, bus shelters and rubbish bins?” categorised 0, 1, 2 or more); truancy (yes/no); cigarette smoking (yes/no); quintiles of equivalised family income; religious affiliation (none vs any of Christian, Muslim, Hindu, Jewish, other).

#### Potential moderating variables

Perception of risk due to alcohol was assessed by the question “How much do you think people risk harming themselves if they drink one or two alcoholic drinks nearly every day?” (no/slight risk, some risk, great risk). Positive expectancies towards alcohol were assessed by the following questions: “Drinking beer, wine, or spirits is a way to make friends with other people”; “It is easier to open up and talk about one's feelings after a few drinks of alcohol”; “Drinking alcohol makes people …worry less; …happier with themselves”. Negative expectancies were assessed using questions: “Drinking alcohol … gets in the way of school work; … makes it hard to get along with friends”; “If I drank alcohol without my parents’ permission I would be caught and punished”. Items were summed and used as two separate scales [23].

Parental supervision was assessed by questions about the weekday and weekend frequency of cohort member spending unsupervised time with friends (playing in the park, going to the shops or just ‘hanging out’). Items were combined into a three category variable: rarely/never (at most occasionally at weekends/on weekdays), sometimes, often (unsupervised most weekends and at least one day per week).

Markers of family relationships were: frequent battles of will with cohort member (yes/no); mother-cohort member closeness (extremely/very close vs fairly/not very close); cohort member happiness with their family (“On a scale of 1 to 7 where ‘1’ means completely happy and ‘7’ means not at all happy, how do you feel about your family?” Responses corresponding to the top decile of the distribution were taken to indicate happy with family) [24].

### Study sample

Data on cohort member drinking were available for 12644 participants. Missing data reduced the sample to 10498 (83.0 %), as follows: friends drinking = 56; puberty = 1010; socioemotional difficulties = 475; religious affiliation = 34; antisocial behaviours = 27; perception of harm = 337; positive expectancies = 189; negative expectancies = 315; parental supervision = 87; frequent battles = 1106; relationship between mother and child = 762; happy with family = 91.

### Statistical analysis

To estimate the association between our exposures of primary interest – mother’s, father’s or friends’ drinking with cohort member drinking, we ran three sets of logistic regression models adding covariates in stages. Boys were more likely to report drinking compared with girls (15.7 vs. 11.3 %), but as there were no gender differences in observed associations between parent and friends’ drinking with cohort member drinking, we present analyses for boys and girls combined, and all models adjust for gender.

Model 0 is the baseline model which includes the primary independent variable (mother’s, father’s or friends’ drinking) and gender.

Model 1 additionally adjusts for control variables: puberty, birth order, socioemotional difficulties, antisocial behaviours, truancy, smoking, income, religion and for other alcohol exposure variables e.g. when mother’s drinking is the primary exposure, we add father’s and friends’ drinking to this step of the analysis.

Model 2 is fully adjusted adding in potential moderator and mediator variables, perception of harm due to alcohol, positive and negative expectancies, parental supervision and family relationships (battles, closeness, happiness with family).

All analysis was carried out using Stata version 13.1 (Stata Corp).

## Results

### Who drinks by age 11?

Overall 13.6 % of cohort members reported having drunk more than a few sips of an alcoholic drink. Cohort members who reported drinking were more likely to be boys (15.7 % vs 11.3 %, *p* < 0.001), to have started puberty (14.3 % vs 13.2 %), to be a second or later born child (14.0 % vs 12.9 %), to have socioemotional difficulties (18.7 % vs 12.8 %, *p* < 0.001), to report antisocial behaviours (none = 10.1 %, 1 = 20.7 %, 2 or more = 42.0 %, *p* < 0.001), report truancy (24.8 % vs 13.2 %, *p* < 0.001), smoke cigarettes (50.9 % vs 12.4 %, *p* < 0.001), to be from poorer families (15.4 % in the poorest quintile vs 11.5 % in richest quintile, *p* < 0.01) and not have any religious affiliation (15.7 % vs 11.6 %, *p* < 0.001). Table 1 shows the distribution of covariates by cohort member drinking.

**Table 1:** Distribution of parent and friends' drinking and covariates by cohort member drinking

	Cohort member drinking	
	No	Yes
	*n* = 9223	*n* = 1275
Mother's drinking		
None	22.9	14.5
Light/moderate	58.9	60.8
Heavy/binge	15.5	21.2
Missing	2.7	3.5
Father's drinking		
None	8.6	5.2
Light/moderate	36.6	34.6
Heavy/binge	17.1	18.9
Father absent from household	27.0	31.9
Missing	10.8	9.4
Friends drink		
No	81.5	56.0
Yes	5.5	25.7
Don't know	13.0	18.3
Child characteristics		
Gender		
Girl	50.5	41.2
Signs of pubertal changes		
Yes	34.7	36.9
Birth order		
Firstborn	42.2	39.8
Socioemotional difficulties		
Yes	12.5	18.3
Anti-social behaviours		
0	79.9	56.9
1	16.8	28.0
2+	3.3	15.0
Truancy		
No	96.3	93.0
Yes	3.0	6.3
No answer	0.7	0.7
Cigarette smoking		
No	98.1	88.9
Yes	1.7	11.0
No answer	0.2	0.2
Perception of harm from 1–2 drinks per day		
No/slight risk	10.9	20.3
Some risk	32.4	38.9
Great risk	56.7	40.8
Alcohol expectancies		
Positive		
0	53.2	36.4
1	22.6	26.2
2+	24.1	37.4
Negative		
3	62.8	43.4
2	32.3	33.0
0 or 1	14.9	23.6
Happy with family		
Yes	89.0	83.0
Family characteristics		
Equivalised household income		
Richest	18.3	21.2
Second	20.7	23.5
Third	20.2	20.9
Fourth	20.3	17.4
Poorest	20.6	17.0
Religious affiliation		
Yes	53.3	44.7
Un-supervised time at weekend/weekday		
Never/rarely	30.9	23.2
Sometimes	30.9	28.5
Often	38.2	48.3
Frequent battles with parents		
Yes	28.0	37.2
Relationship between mother and child		
Very/extremely close	93.6	91.1

### Does parental or friends’ drinking matter?

Cohort members whose mothers drank were more likely to drink and these estimates changed little on adjustment for covariates (fully adjusted OR – light/moderate = 1.6, 1.3 to 2.0, heavy/binge = 1.8, 1.4 to 2.3 compared to those with non-drinking mothers). Cohort members for whom data on mother’s drinking was missing were also more likely to drink (fully adjusted OR = 2.0, 1.2 to 3.4). Cohort members whose fathers drank were also more likely to drink but these estimates lost statistical significance when covariates were taken into account (fully adjusted OR – light/moderate = 1.3, 0.9 to 1.9, heavy/binge = 1.3, 0.9 to 1.9). Having friends who drank was associated with more 7 times the odds of cohort member drinking, and twice the odds when cohort members reported not knowing whether their friends drank. These estimates changed on adjustment for covariates but remained highly statistically significant (fully adjusted ORs 4.8, 3.9 to 5.9 and 1.8, 1.4 to 2.2 respectively) (Table 2).

**Table 2:** Odds (95 % CI) of cohort member drinking

	Model 0		Model 1		Model 2	
Mother's drinking (ref None)						
Light/moderate	1.64***	(1.35 to 1.98)	1.71***	(1.39 to 2.09)	1.59***	(1.29 to 1.96)
Heavy/binge	2.17***	(1.73 to 2.71)	1.93***	(1.49 to 2.49)	1.78***	(1.37 to 2.32)
Missing data on drinking	2.09**	(1.29 to 3.38)	2.21**	(1.32 to 3.70)	2.02**	(1.19 to 3.44)
Father's drinking (ref None)						
Light/moderate	1.55**	(1.12 to 2.14)	1.40	(0.99 to 1.98)	1.32	(0.92 to 1.88)
Heavy/binge	1.83***	(1.32 to 2.53)	1.43*	(1.00 to 2.05)	1.31	(0.90 to 1.90)
Father absent from household	1.93***	(1.40 to 2.66)	1.26	(0.90 to 1.77)	1.16	(0.82 to 1.65)
Missing data on drinking	1.44*	(1.00 to 2.05)	1.10	(0.75 to 1.62)	1.03	(0.70 to 1.51)
Friends drink (ref No)						
Yes	6.75***	(5.59 to 8.15)	5.23***	(4.25 to 6.43)	4.80***	(3.91 to 5.88)
Don't know	2.04***	(1.68 to 2.48)	1.88***	(1.52 to 2.32)	1.78***	(1.44 to 2.19)

Model 0 adjusts for gender

Model 1 - Model 0 + puberty, birth order, socioemotional difficulties, income, religion, anti-social behaviour, truancy, cigarette smoking

Model 2 - Model 1 + perception of harm due to alcohol, positive and negative expectancies, parental supervision, battles, closeness, happiness with family

*** *p* < 0.001, ** *p* < 0.01, * *p* < 0.05

### What is the role of perceptions of harm, expectancies towards alcohol, parental supervision, and family relationships?

Perceptions of harm, expectancies towards alcohol, parental supervision, and family relationships were associated with the likelihood of cohort member drinking in the expected direction (Appendix Table 3). Associated with the reduced likelihood of cohort member drinking were: heightened perception of harm from drinking 1–2 drinks daily (OR - some risk = 0.9, 0.7 to 1.1, great risk = 0.6, 0.5 to 0.7); and negative expectancies towards alcohol (OR = 0.5, 0.4 to 0.7). Associated with an increased risk of cohort member drinking were: positive expectancies towards alcohol (OR = 1.9, 1.4 to 2.5); not being supervised by parents on weekends and weekdays (for often OR = 1.2, 1.0 to 1.4); frequent battles of will (OR = 1.3, 1.1 to 1.5); and not being happy with family (OR = 1.2, 1.0 to 1.5).

## Discussion

Our results suggest that nearly 14 % of 11 year olds in the UK have had an alcoholic drink. The odds of drinking were greater when their friends drank compared to when their parents drank: boys and girls who reported having friends who drank were five times more likely to report drinking themselves compared to those who reported having friends who did not drink. Having a mother who drank heavily was associated with an 80 % increased odds of drinking, however, fathers’ drinking was not independently associated with children’s drinking. Our results suggest that 11 year olds’ perceptions of risk, their expectancies towards alcohol and relationships with their families were independently related to the likelihood of drinking.

Distinct strengths of this work are that we used data from a large sample representative of 11 year olds in the UK; we simultaneously examined relationships with parents and friends drinking; and we were able to take into account rich contextual information about young people’s understanding of the risk of drinking alcohol, their expectancies, positive and negative, towards alcohol and family relationships. On the other hand, there are several limitations to acknowledge, including that the analyses were cross sectional as information on cohort member and friends' drinking, perceptions of harm and expectancies around drinking are only available from one wave of data collection thus causal inference cannot be drawn; the data on cohort member and friends’ drinking were developed for the MCS survey making it difficult to compare prevalence rates with other studies, although closed questions as used in this study have been shown to be valid markers of alcohol consumption in adolescents [25]; the data on cohort member and friends’ drinking were reported by the cohort member and thus may be prone to under or over estimation with one prior contemporary study suggesting a lower prevalence of drinking among British 11 year olds [10], although this may be due to different survey questions; we were not able to distinguish those who had just tried one or two drinks ever from cohort members who are regularly drinking; also there were no data available on the context of cohort member drinking and so it was not possible to assess the circumstances in which, or with whom, 11 year olds drank.

Prior work has charted the prevalence of drinking among 11 year olds in the UK [10] and elsewhere [26, 27]. To our knowledge this is the first UK study in this young age group to attempt a detailed exploration of family and peer influences, along with the young person’s views about alcohol on the likelihood of drinking. Moreover, most prior work has been set in the US [28] and it may be that associations vary across contexts [9]. We examined associations between parent and friends’ drinking and family relationships at the very start of the adolescent period, whereas prior studies have looked at these associations among older adolescents. For instance, Cable and Sacker’s examination of 16 year olds from the 1970 Birth cohort suggests that negative expectancies are not protective [13]. However, we might expect to see the same pattern of association as adolescence proceeds with peer influences and associated social norms having a more profound effect on alcohol use in later than early adolescence [13, 15–17].

A recent Cochrane review [29] concluded there was limited evidence that school/education based intervention programmes were effective, and where they did work the focus was more holistic, not solely on alcohol. In keeping with this we found markers of other risky behaviours, including smoking and antisocial behaviours to be strongly independently related to drinking at age 11. Clearly, there are opportunities to intervene and help shape choices around risky behaviours including drinking. Our findings support policies working at multiple levels that incorporate family and peer factors. For example: compared with mother’s drinking, father’s drinking was not as strongly related to drinking in their 11 year olds but this may be because fathers are more likely to drink in settings other than the home. Our observations that greater awareness of the harms from alcohol and negative expectancies are associated with reduced odds of 11 year olds drinking support strategies to empower young people to say no to alcohol. This is particularly important, as undoubtedly, peer influences become stronger in shaping young people’s behaviours as adolescence proceeds.

Our study was not able to examine contexts around drinking occasions among 11 year olds – who do they drink with? Where, when and what do they drink? How do they acquire alcohol and what are the broader social norms around drinking? One study that compared young people’s drinking in Italy and Finland showed that Italian youth were more likely to drink with meals under family supervision, whereas Finnish youth were more likely to drink in settings that led to drunkenness [9]. Being able to investigate context in more detail would help inform alcohol harm prevention strategies. Longitudinal studies looking at changes in expectancies towards alcohol and how these relate to changes in young people’s behaviours including potential clustering with other risky behaviours are important areas for future study.

## Conclusions

Examining drinking at this point in the lifecourse has potentially important public health implications as around one in seven 11 year olds have drank, although the vast majority are yet to explore alcohol. Even though the links between early drinking and later life drinking problems remain unclear, we need to further improve our understanding of the relative importance and meaning of drinking in early adolescence as regular and heavy drinking among young people is linked to harmful behaviours and premature death. However, apparent culturally specific differences in the meaning of drinking underscores the importance of identifying factors that shape early drinking experiences across settings. Improving our understanding of context specific drivers of early drinking presents golden opportunities to develop effective policy and prevention strategies.

## Ethical approval

Ethical approval was not required for secondary analysis of publically available archived data.

## Appendix

**Table 3:** Odds (95 % CI) of cohort member drinking

	Model 0		Model 1		Model 2	
Gender						
Girl	0.68***	(0.60 to 0.77)	0.67***	(0.58 to 0.78)	0.68***	(0.58 to 0.79)
Signs of pubertal changes						
Yes			1.38***	(1.17 to 1.63)	1.40***	(1.19 to 1.65)
Birth order						
Firstborn			0.93	(0.80 to 1.10)	0.91	(0.77 to 1.08)
Socioemotional difficulties						
Yes			0.97	(0.79 to 1.20)	0.80	(0.63 to 1.00)
Equivalised household income (ref poorest)						
Fourth			0.95	(0.74 to 1.22)	0.99	(0.77 to 1.27)
Third			0.97	(0.73 to 1.27)	1.00	(0.76 to 1.32)
Second			0.90	(0.67 to 1.21)	0.94	(0.70 to 1.25)
Richest			0.89	(0.66 to 1.18)	0.92	(0.69 to 1.24)
Religious affiliation						
Yes			0.81**	(0.69 to 0.94)	0.84*	(0.72 to 0.99)
Anti-social behaviours (ref 0)						
1			1.87***	(1.56 to 2.25)	1.73***	(1.43 to 2.09)
2			3.22***	(2.13 to 4.87)	2.80***	(1.85 to 4.23)
3			3.67***	(2.04 to 6.60)	2.92***	(1.64 to 5.18)
4			5.14**	(1.81 to 14.60)	3.92**	(1.40 to 11.0)
Truancy (ref No)						
Yes			0.93	(0.63 to 1.38)	0.86	(0.57 to 1.28)
No answer			1.07	(0.31 to 3.71)	1.11	(0.34 to 3.60)
Cigarette smoking (ref No)						
Yes			3.23***	(2.26 to 4.60)	3.03***	(2.17 to 4.24)
No answer			0.53	(0.12 to 2.33)	0.51	(0.15 to 1.79)
Perception of harm from 1–2 drinks per day (ref No/slight risk)						
Some risk					0.88	(0.70 to 1.10)
Great risk					0.59***	(0.47 to 0.74)
Alcohol expectancies						
Positive					1.87***	(1.42 to 2.46)
Negative					0.51***	(0.39 to 0.66)
Un-supervised time at weekend/weekday (ref Never/rarely)						
Sometimes					1.03	(0.85 to 1.26)
Often					1.15	(0.95 to 1.38)
Frequent battles with parents						
Yes					1.25*	(1.05 to 1.50)
Relationship between mother and child (ref Very/extremely close)						
Fairly/not very close					1.30	(0.99 to 1.71)
Happy with family (ref Yes)						
No					1.24*	(1.02 to 1.52)

Model 0 adjusts for gender

Model 1 - Model 0 + puberty, birth order, socioemotional difficulties, income, religion, anti-social behaviour, truancy, cigarette smoking

Model 2 - Model 1 + perception of harm due to alcohol, positive and negative expectancies, parental supervision, battles, closeness, happiness with family

*** *p* < 0.001, ** *p* < 0.01, * *p* < 0.05

## Abbreviations

CI: confidence interval
MCS: Millennium Cohort Study
OR: odds ratio
UK: United Kingdom
