# Erratum to: What influences 11-year-olds to drink? Findings from the Millennium Cohort Study

**DOI:** 10.1186/s12889-016-3501-3

**Published:** 2016-08-18

**Authors:** Yvonne Kelly, Alice Goisis, Amanda Sacker, Noriko Cable, Richard G. Watt, Annie Britton

**Affiliations:** 1 Department of Epidemiology and Public Health, University College London, London, WC1E 6BT UK; 2 Department of Social Policy, The London School of Economics and Political Science (LSE), Houghton Street, London, WC2A 2AE UK

## Erratum

In the publication of this article [1], the Acknowledgements section failed to include the following:

“The alcohol use and attitudes variables in MCS5 were co-funded by grant AA013606 from the U.S. National Institute on Alcohol Abuse and Alcoholism”.
